# A Modification to a Murine Model for Intracranial Aneurysm Formation and Rupture

**DOI:** 10.7759/cureus.16250

**Published:** 2021-07-08

**Authors:** Devan Patel, William S Dodd, Kartik Motwani, Koji Hosaka, Brian L Hoh

**Affiliations:** 1 Department of Neurosurgery, University of Florida, Gainesville, USA

**Keywords:** aneurysm formation, aneurysm rupture, animal model, intracranial aneurysm, cerebral aneurysm

## Abstract

Between 3.6% and 6.0% of the population has an intracranial aneurysm. The mechanisms underlying intracranial aneurysm formation and rupture are not fully known. Several rodent models have been developed to better understand intracranial aneurysm pathophysiology. Hypertension, hemodynamic changes, and vessel injury are all necessary for aneurysm induction; however, multiple invasive procedures may disrupt an animal’s physiology. Therefore, we hypothesized that our method for inducing hypertension could be modified to create a simpler model. We previously developed a highly reproducible murine model of intracranial aneurysm formation and rupture that involves hemodynamic changes through ligation of the left common carotid artery, vessel wall degradation using elastase and a lysyl oxidase inhibitor, and hypertension through a high-salt diet, continuous angiotensin II infusion, and right renal artery ligation. In order to create a simpler model, we sought to eliminate renal artery ligation. We assessed aneurysm formation, aneurysm rupture, and blood pressure in two separate cohorts of C57BL/6 mice: one cohort underwent our model as above, while another cohort did not receive right renal artery ligation. Our results demonstrate that intracranial aneurysm formation and rupture rates did not differ between each group. Further, the blood pressures between cohorts did not differ at various timepoints in the model. Both cohorts, however, did have a significant increase in blood pressure from baseline, suggesting that renal artery ligation is not needed for inducing hypertension. These findings demonstrate that our murine model can be modified to eliminate right renal artery ligation. Thus, we propose this modification to our murine model for studying intracranial aneurysm pathophysiology.

## Introduction

Intracranial aneurysms (IA) affect between 3.6-6.0% of the population [1]. IA rupture results in subarachnoid hemorrhage, a devastating condition with up to 51% mortality [2]. The pathophysiology of IA formation and rupture is not fully understood and various rodent models have been developed to investigate underlying mechanisms [3-12].

We previously described a highly reproducible murine model for IA formation and rupture [5]. This model includes hemodynamic changes, hypertension, and vascular wall degradation, all key features of human IA [13]. Briefly, mice underwent two sets of procedures: first, the right renal artery (RRA) and left common carotid artery (LCCA) are ligated to induce hypertension and hemodynamic changes, respectively. Then, one week later, elastase is stereotaxically injected into the basal cisterns to facilitate vascular wall degradation, and an angiotensin II osmotic pump is implanted to further induce hypertension. At the time of this second procedure, mice are fed a diet containing high salt content (8% sodium chloride) and β-aminopropionitrile (BAPN, 0.12%), a lysyl oxidase inhibitor that facilitates vessel wall degradation [5,14]. This methodology results in IA formation and rupture rates of 67-90% and 50-61%, respectively [5,15]. While this murine model is highly reproducible, we believe the model can be simplified to have fewer confounding effects on the mouse’s baseline physiology.

We sought to modify our current murine IA model while maintaining reproducibility and clinical applicability. Given that our model uses several methods of inducing hypertension, we investigated if RRA ligation is necessary. Renal ischemia, as induced by renal artery ligation, increases angiotensin II which will subsequently result in elevated blood pressures [16]. In our established model, we hypothesize that renal artery ligation is an unnecessary, invasive procedure, given that the model already includes the implantation of an angiotensin II infusion pump. Removing this step while preserving key elements of the model would result in improved animal welfare and a simpler model. Thus, the following study aims to determine whether renal artery ligation is necessary for murine models of IA formation and rupture.

## Materials and methods

Animals

Animal experiments were conducted with approval from the University of Florida Institutional Animal Care and Use Committee and in accordance with the ARRIVE (Animal Research: Reporting of In Vivo Experiments) guidelines. Animals were housed in a normal specific pathogen-free room with food and water ad libitum and 12-hour light/dark cycling.

Murine intracranial aneurysm model

8-10-week-old C57BL/6 female mice (Charles River, Wilmington, Massachusetts, USA) were used for all experiments. Female mice were used, given both our previous experiences with this model and human epidemiological studies, which suggest females may be more affected than males by IAs [1-2,5]. Mice were randomly assigned to undergo either LCCA ligation and RRA ligation (“established model”, n=12) or LCCA ligation only (“modified model”, n=12). Sample sizes were based on prior studies with our established model [5]. For all procedures, anesthesia was induced via intraperitoneal injection of ketamine and xylazine. Ligations were performed using 8-0 nylon suture (Ethicon, Somerville, New Jersey, USA). Two weeks later, elastase [10 uL of 1 unit/mL elastase in phosphate-buffered saline (PBS)] (Worthington Biochemical Corporation, Lake Wood, New Jersey, USA) was injected into the right basal cistern using a stereotaxic frame with a mouse adaptor (Stoelting, Wood Dale, Illinois, USA). Coordinates for the elastase injection were 1.2 mm rostral to bregma, 0.7 mm lateral to the midline, and 5.0 mm deep to the surface of the brain, as previously described [5]. Mice were then implanted with a subcutaneous osmotic pump (Durect, Cupertino, Massachusetts, USA) for continuous infusion of angiotensin II (BACHEM, Torrance, California, USA) in PBS (1000 ng/kg/min). After the elastase injection and osmotic pump implantation, mice were started on a diet containing 8% sodium chloride and 0.12% β-aminopropionitrile (Harlan Laboratories, Indianapolis, Indiana, USA). 

Animals were monitored for signs of aneurysm rupture, such as circling paresis and inactivity. If present, mice were euthanized via cardiac perfusion of PBS followed by 4% paraformaldehyde (Sigma-Aldrich, St Louis, Missouri, USA). The circle of Willis was assessed for aneurysm formation and/or rupture using perfusion of Coomassie Brilliant Blue dye (Thermo Scientific, Waltham, Massachusetts, USA) dissolved in 15% (w/v) gelatin (Thermo Scientific, Waltham, Massachusetts, USA) in PBS. The remaining mice were euthanized 21 days post-elastase injection. Aneurysms were identified as an outpouching of a cerebral artery in or around the circle of Willis. Aneurysm rupture was identified by the presence of extravasated blood. 

Blood pressure measurements

A noninvasive CODA tail-cuff monitor (Kent Scientific, Torrington, Connecticut, USA) was used for blood pressure measurements. Five readings were measured for each mouse at the following time points: baseline prior to ligation(s), one week after ligation(s), and one week after elastase injection. Blood pressures are reported as average mean arterial pressure (MAP) ± standard error of the mean.

Blinding and randomization

Animals were randomly assigned to each experimental group. Blood pressure measurements and assessment of aneurysm formation and rupture were performed in a blinded manner.

Statistical analysis

Differences in aneurysm formation and rupture were identified using Fisher’s exact test. Differences in blood pressure were assessed using the Mann-Whitney U test. The threshold for statistical significance was p<0.05. 

## Results

Intracranial aneurysm formation and rupture

**Figure 1:**
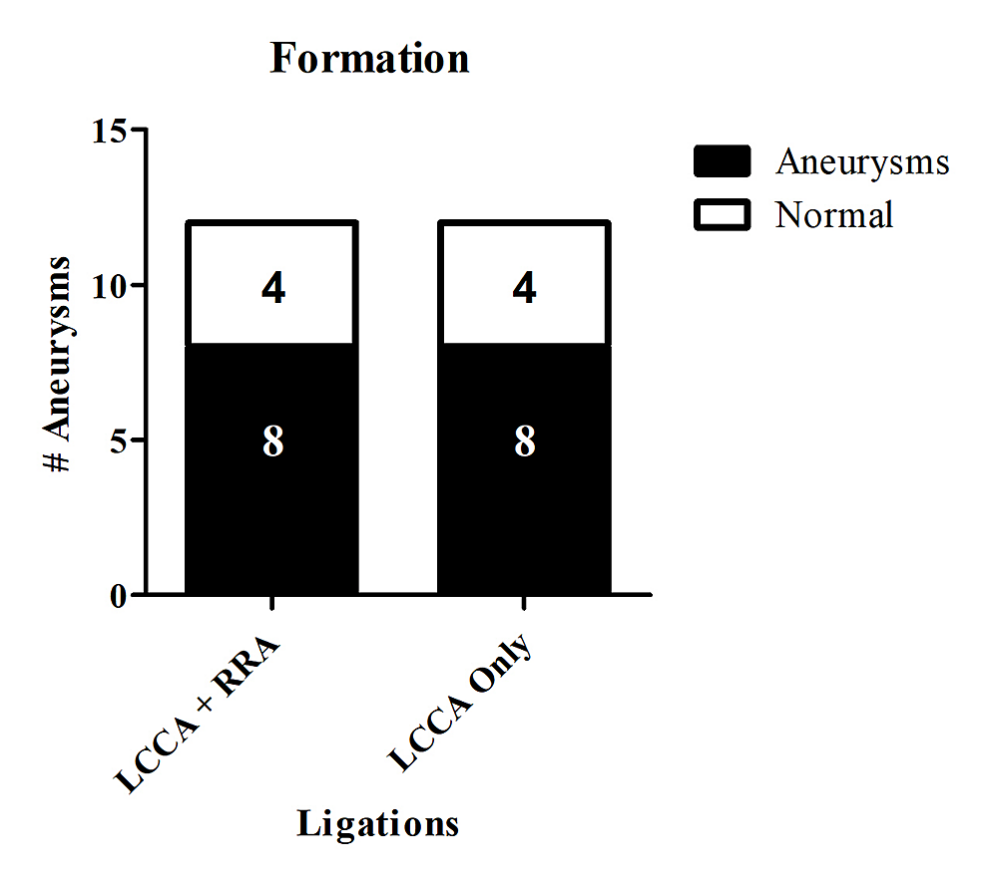
Intracranial aneurysm formation Performing LCCA ligation only does not affect aneurysm formation compared to ligating both the LCCA and RRA. Aneurysm formation was 66.7% in both groups (p=1.00). LCCA = left common carotid artery, RRA = right renal artery.

**Figure 2:**
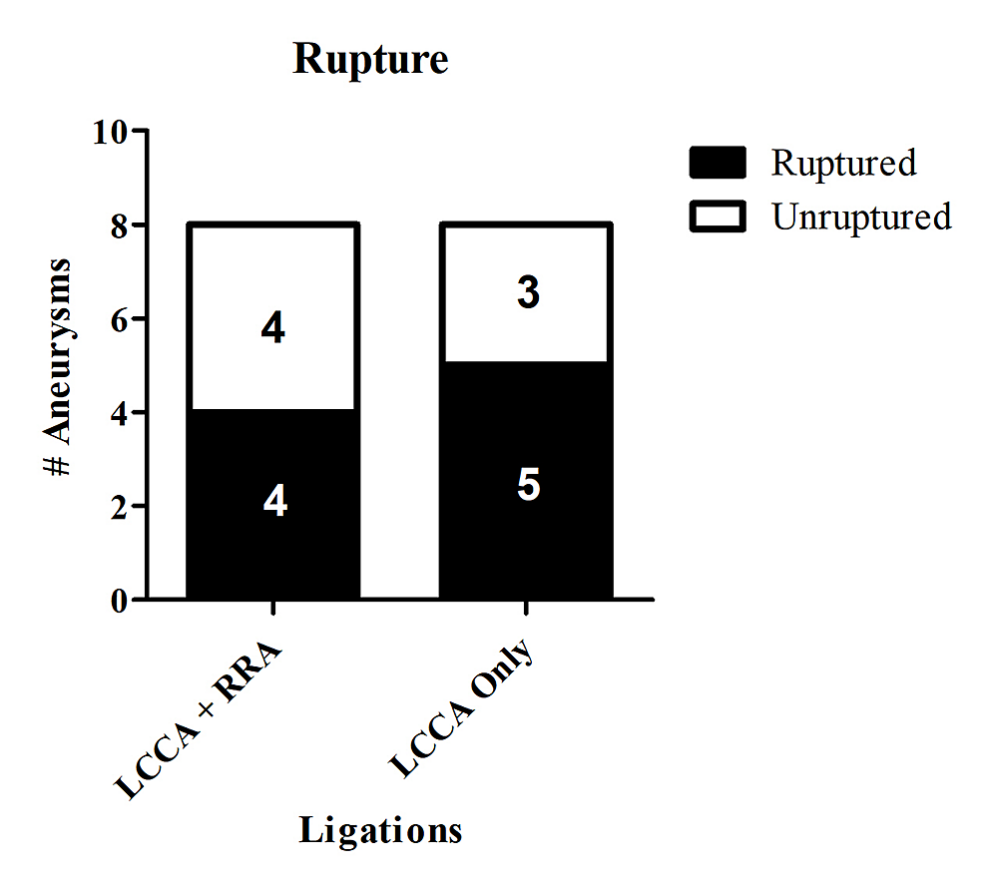
Intracranial aneurysm rupture Only ligating the LCCA does not affect aneurysm rupture compared to ligating both the LCCA and RRA. In the LCCA ligation-only group, 62.5% of aneurysms were ruptured compared to 50% of aneurysms ruptured in the LCCA+RRA ligations group (p=1.00). LCCA = left common carotid artery, RRA = right renal artery.

The modified model, which does not include RRA ligation, did not affect aneurysm formation or rupture compared to the established model (aneurysm formation: LCCA ligation only 66.7% versus LCCA+RRA ligations 66.7%, p=1.00; rupture: LCCA ligation only 62.5% versus LCCA+RRA ligations 50.0%, p=1.00) (Figures 1, 2).

Blood pressure

**Figure 3:**
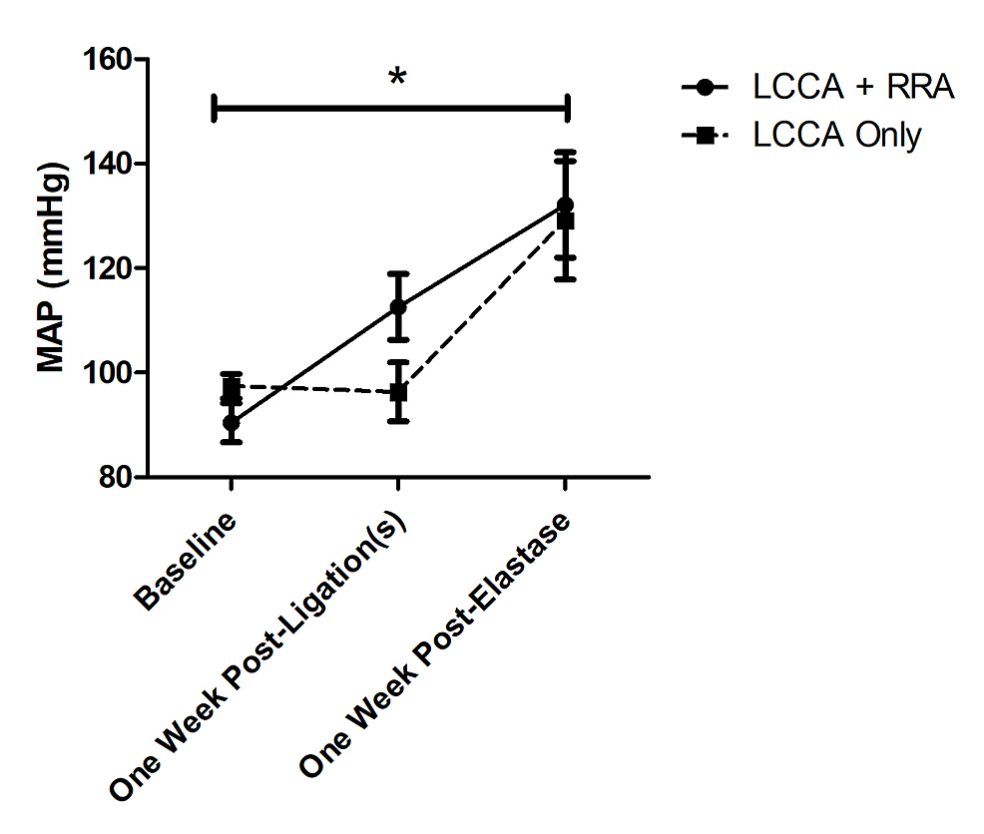
Blood pressure The average MAP of mice in the LCCA ligation only group did not differ significantly from the average MAP of mice in the LCCA+RRA ligations group at any time point (baseline: 97.4±2.34 mmHg versus 90.4±3.73, p=0.22; one-week post-ligation(s): 96.3±5.64 mmHg versus 112.6±6.28 mmHg, p=0.15; one-week post-elastase: 129.2±11.3 mmHg versus 132.1±10.0 mmHg, p=1.00). The average MAP in both groups increased significantly from baseline to one-week post-elastase (LCCA ligation only: 97.4±2.34 mmHg versus 129.2±11.3 mmHg, p=0.02; LCCA+RRA ligations: 90.4±3.73 mmHg versus 132.1±10.0 mmHg post-elastase, p=0.04), indicating that RRA ligation is not required to induce hypertension. MAP = mean arterial pressure, LCCA = left common carotid artery, RRA = right renal artery.

The modified model, which does not include RRA ligation, did not affect MAP compared to the established model at any of the time points measured. At baseline prior to ligation(s), the MAP in the LCCA ligation only group was 97.4±2.34 mmHg and in the LCCA+RRA ligations group was 90.4±3.73 mmHg (p=0.22). One week after ligation(s), the MAP in the LCCA ligation only group was 96.3±5.64 mmHg and in the LCCA+RRA ligations group was 112.6±6.28 mmHg (p=0.15). One week after elastase injection, the average MAP was 129.2±11.3 mmHg in the LCCA ligation only group and 132.1±10.0 mmHg in the LCCA+RRA ligations group (p=1.00)(Figure 3). 

In both groups, the MAP did significantly increase from baseline measurements to post-elastase injection measurements. In the LCCA ligation-only group, the average MAP increased from 97.4±2.34 mmHg at baseline to 129.2±11.3 mmHg (p=0.02). In the LCCA+RRA ligation group, the average MAP increased from 90.4±3.73 mmHg at baseline to 132.1±10.0 mmHg post-elastase (p=0.04) (Figure 3).

## Discussion

The precise mechanisms involved in IA formation and rupture are unknown. Although human tissue samples are useful in understanding fundamental concepts about IA, many critical features, such as early inflammatory processes leading to aneurysm formation and rupture, cannot be studied as a surgical collection of tissues serves as an artificial endpoint to IA pathophysiology. Thus, a reproducible animal model of both IA formation and rupture is crucial to achieving a better understanding and developing novel therapeutic options.

While several models have been used to study IA formation, there are a few models that reproducibly result in IA rupture [3,7,11]. Mouse models are particularly invaluable given the utility of genetic modifications [12]. We previously reported a murine model of aneurysm formation and rupture using elastase, hemodynamic changes, and hypertension [5]. While this model has been used to uncover novel mechanisms and therapeutic targets, we sought to modify the model to be simpler [15,17-19]. 

We and others have shown a combination of elastase, hemodynamic changes, and hypertension are necessary to reliably study IA formation and rupture while maintaining key features of human IA [3,5,7,11]. While elastase injection and ligation of the LCCA are required for IA formation and rupture, there are several options for inducing hypertension [3,7,11,12]. In addition to a high-salt diet, our established model increases angiotensin II via RRA ligation and continuous infusion of angiotensin II. Here, we hypothesized that eliminating RRA ligation will not affect IA formation and rupture rates and is not necessary for the development of hypertension.

Indeed, RRA ligation can be eliminated from our model. In mice that underwent LCCA ligation only, the IA formation rate was identical to the cohort that received both LCCA and RRA ligations. Similarly, the IA rupture rate was not significantly different between the LCCA ligation-only cohort and the LCCA+RRA ligation cohort. 

As hypertension is a key component of IA pathophysiology, we evaluated whether RRA ligation was necessary to increase blood pressure in our model [13]. MAP did not differ between the LCCA ligation-only cohort and the LCCA+RRA ligation cohort at baseline, post-ligation(s), or post-elastase injection. When comparing baseline MAP to post-elastase MAP, there is a significant increase in blood pressure in both groups, suggesting that continuous angiotensin II infusion and a high-salt diet are sufficient for inducing hypertension.

## Conclusions

The results of this study demonstrate that RRA ligation was not necessary in our murine model of IA formation and rupture. Continuous infusion of angiotensin II via an osmotic pump and a high-salt diet are sufficient to induce hypertension in mice. Eliminating RRA ligation results in a model that is simpler, more humane, and less invasive. Nonetheless, the modified model proposed here maintains key components of IA animal models, namely hypertension, hemodynamic changes, and vessel wall degradation. Further, the advantages of this murine model over other models, including higher and more consistent rates of IA formation and rupture, are preserved.
